# Inverted Microstrip Gap Waveguide Filtering Antenna Based on Coplanar EBG Resonators

**DOI:** 10.3390/s23010282

**Published:** 2022-12-27

**Authors:** Luis Inclán-Sánchez

**Affiliations:** Department of Signal Theory and Communications, Universidad Carlos III de Madrid, 28911 Leganés, Spain; linclan@ing.uc3m.es

**Keywords:** filtering antenna, Gap Waveguide Technology, interference mitigation, slot antenna, inverted microstrip, electromagnetic bandgap structure, stopband filter, antenna array, feeding network, X-band

## Abstract

A new simple design of an inverted microstrip Gap Waveguide filtering antenna integrated with two stopband filters is proposed in this work. In order to simultaneously provide filtering and radiating functions, we use the direct integration approach to cascade two periodic sets of coplanar coupled EBG resonators with a slot antenna. The analysis shows that the filters can be easily adjusted in the same feeding layer of the antenna, without extra circuitry and without modifying the lines. EBG-filters are compact and offer great flexibility in determining the frequency, width and selectivity of the rejected bands. Experimental results for an X-band filtering antenna prototype are provided showing a 7.3% transmission band centered at 10.2 GHz and a realized gain peak of 2.1 dBi. The measurements demonstrate the filtering capability of the proposed antenna, achieving rejection levels greater than 12 dB and 20 dB for the bands below and above the operation band. The proposed low-complexity design offers good performance as a filter and as an antenna, showing the essential advantages of the Gap Waveguide Technology, including low losses, self-packaging and limited cost. This work demonstrates the possibility of integrating the new coplanar EBG-filters into future Gap Waveguide antenna designs to avoid unwanted radiation, to reduce interfering signals or to provide high isolation in multiband systems.

## 1. Introduction

The increasingly demanding radio environment with critical saturation, noise and interference conditions implies that radio frequency (RF) front-end systems need to be innovated to manage advanced wireless systems [1,2]. These systems could operate applications that include communications, sensing and monitoring to provide a very high data rate, low latency and an efficient use of spectral and energy resources [3,4,5]. In this radio environment, there is no doubt that antennas and filters, always necessary in RF front-ends, play a crucial role in efficiency and final system performance, as in [6]. RF-systems, for sensing and communications applications, degrade their behavior due to interference and coupling. Filters and electromagnetic bandgap structures (EBG) have been used for interference mitigation. Among other things, they have been designed to avoid unwanted radiation, cancel radiation at harmonic frequencies and mitigate adjacent channel interference. In this context, some technologies have emerged with the aim of improving some of these characteristics. For example, the substrate integrated waveguide technology (SIW) competes with others by offering cheaper printed circuit board (PCB) technology for designs with higher levels of integration [7]. Another interesting example is the so-called Gap Waveguide Technology (GWG) [8]. This technology offers various advantages, among which its low losses and self-packaging stand out. In particular, we are going to focus on the inverted microstrip Gap Waveguide (IM-GWG) version in which the antenna object of this study is proposed [9]. In this printed version of the technology, it has been demonstrated that numerous antennas obtain a remarkable performance [10,11].

In addition to the defined technological aspects (such as provided by PCB, SIW or GWG) and suitable electromagnetic operation, devices for modern wireless systems must fulfill other requirements such as low cost, space restrictions, energy efficiency and mechanical robustness. Antenna engineering has long been developing the concept of filtering antenna (also called filtenna) [12,13]. These devices consist in the integration of filters together with the radiating elements so that the resulting device simultaneously performs the functions of filtering and radiation [12]. The main advantage that these systems have is that they eliminate the filter as an independent element, saving space in the device and improving energy efficiency. Of course, the goal of the design of these filtering antennas is to provide an integrated operation, in filtering and radiant performance, as good as or even better than the independent elements. For all these reasons, filtering antennas have attracted extensive research efforts. Many types of these kinds of antennas and their design methods have been reported in the state of the art [12,13], including also the possibility of making them reconfigurable [14]. We are going to group a set of filtering antenna designs into four methodologies, the first three correspond to typical approaches and the fourth to more recent techniques. The first design method we find in the literature is direct integration. In this case, the antenna is individually designed and integrated with a bandpass filter. The filter and the antenna are cascaded, through 50 Ω reference impedances, to select the desired passband and suppress the undesirable signal out of the band. Examples of this approach are [15,16,17,18,19,20,21] on microstrip or PCB technology and [22,23] on waveguide technology, while [24] feature an SIW-based design. The second set of works correspond to the so-called co-design method. In this case the antenna is used simultaneously as a radiating element and as part of the filter. That is, the filter is synthesized using the antenna as the last stage resonator and load impedance. In [25], this methodology is illustrated; other examples are [26,27,28,29] in microstrip and [30,31,32] for SIW technology. Broadening the concept of filtering antenna, we can group it into a third methodology, called fusion method in [12], which includes antennas that do not incorporate an extra filtering circuit. In this strategy, resonant structures are integrated in parallel with the antenna to obtain stopband functions at both sides of the transmission/radiation band. Another similar option is to modify the antenna to avoid mode excitation and produce radiation nulls close to the band edges. In any case, the purpose is always to control these radiation nulls to generate the passband and tune the frequency selectivity of the antenna and the out-of-band rejection. We find numerous examples in the literature such as [33,34,35,36,37,38,39]. Finally, we group in a fourth category more recent works that use new techniques to produce the filtering of the antenna, such as the metasurface concept [40] or the spoof surface plasmon polaritons [41].

Focusing on Gap Waveguide Technology, some antenna designs that integrate filters have been published. A full-duplex RF-front end operating in the E-band has been demonstrated in [42] and an integrated diplexed-antenna in the Ka-band in [43], in both cases, a high-gain planar array of slots is integrated with groove Gap Waveguide filters and with diplexer splitters. There are also other designs in GWG that use filters for diplexer applications, in the groove version [44] and in the inverted microstrip [45], although none have been integrated with antennas. There are few works in GWG in which an explicit design of filtering antenna is described. Some have recently been published offering preliminary designs using this concept. An initial numerical study of the work described in this article in the inverted microstrip version is presented in [46], while in [47,48] two designs made in the groove version have been described.

Considering the challenge of RF-interference control, the aim of the work is to add filtering capabilities to GWG antennas, with low complexity and high isolation/rejection properties. Consequently, in this paper, based on the preliminary work in [46], an inverted microstrip GWG filtering antenna using EBG resonators is proposed. The antenna incorporates two filters based on [49], which allow rejecting two bands that can be flexibly adjusted. These filters are easily integrated with a slot antenna that simultaneously achieves filtering and radiation functionality. The EBG filters have coplanar resonators and short circuit elements, so they are implemented in the same layer as the antenna’s feeding line. This allows its integration in a simple way and without extra space requirements, taking advantage of the IM-GWG antenna feeding networks. This filtering antenna allows working in higher frequency bands, from X-band to mmWave band and beyond, reducing losses, without electrical contact between layers and incorporating self-packaging. The study details the experimental characterization of the new dual-band filter and the antenna. The measurements demonstrate the efficient radiation of the filtering antenna in the pass band and the high rejection levels obtained in the out-of-bands.

The document is organized as follows. Designs for the dual band filter, slot antenna and filtering antenna are detailed in Section 2. Section 3 includes the description of the manufactured prototypes, the filter and the two antennas and their experimental characterization. A discussion of the results and their comparison with the state of the art is also included at the end of Section 3. Finally, Section 4 summarizes the main conclusions of the work.

## 2. Filtering Antenna Design

**Figure 1:**
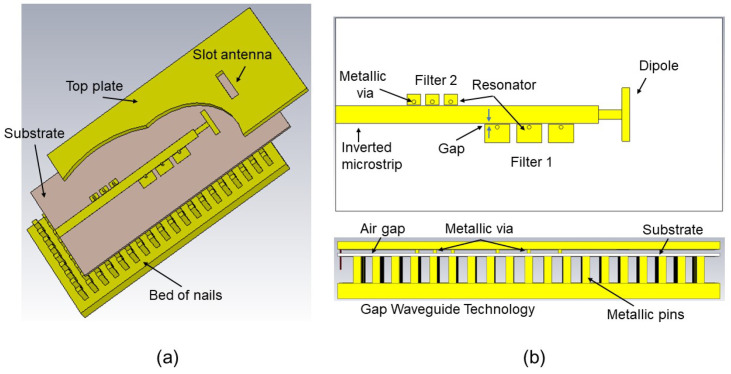
Filtering antenna topology in IM-GWG. (**a**) 3D view of the multilayer configuration. (**b**) Top view of the printed circuit layer and side view of the GWG.

This section describes the design methodology of the filtering antenna. The expected behavior of the new device will also be shown through simulations. In this work, all the simulation analyses have been carried out with the CST Microwave Studio. The filters used in the work are designed from [49]. The configuration of the IM-GWG is identical to the one analyzed in [49] and has the same basic geometric parameters. The proposed IM-GWG filtering antenna is depicted in Figure 1, which is mainly composed of three layers. The bottom layer is the full metal square array of pins that makes up the artificial surface named the bed of nails. This surface behaves like a perfect magnetic conductor in a frequency range, enabling the operation of the Gap Waveguide. As shown in Figure 1a, the upper layer of the GWG consists of an aluminum plate in which the rectangular slot antenna is made. In the middle of these two layers is the substrate on which the inverted microstrip feeding line and the EBG resonators are printed as Figure 1b shows. The substrate is a Rogers 4003 with thickness hs = 0.79 mm and permittivity $\epsilon_{r}=3.38$. The manufacturer characterizes the material losses with a tgδ=0.0027 at a frequency of 10 GHz. Between the substrate and the top cover of the waveguide, there is a 1mm thick air gap, as shown in Figure 1b. This distance, between PMC and PEC layers, is much smaller than λ/4, so there is no possibility of exciting parallel plate electromagnetic modes. In the space between the microstrip line and the upper part, this condition is broken, allowing the local propagation of the QTEM mode. In essence, electromagnetic energy propagates in air, which is why the GWG technology allows the signal to propagate with low losses even at high frequencies.

### 2.1. Integrated Filtering Antenna Concept

**Figure 2:**
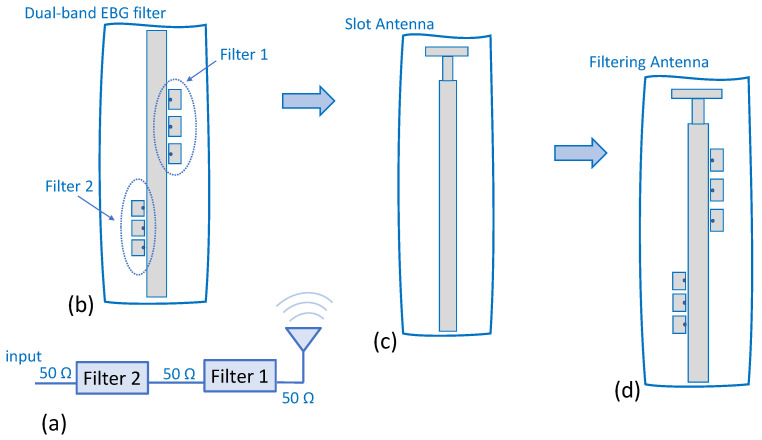
Description of the IM-GWG cascaded two filters and slot antenna. (**a**) Schematic. (**b**) Dual-band EBG filter. (**c**) Slot antenna. (**d**) Filtering antenna.

The design mechanism of the IM-GWG filtering antenna is derived from the simplest method of direct integration [12]. On the one hand, this typical solution involves independent designs of the filter and the antenna. On the other hand, it is necessary to connect them properly to tune the frequency response with the system requirements. In our case, we propose to connect two stopband filters and the antenna through a 50 Ω impedance interface, as shown in the schematic included in Figure 2a. The conceptual evolution from the slot radiator (Figure 2c) to the filtering antenna with the two EBG filters is shown in Figure 2. As can be seen in Figure 2a, the two EBG-filters are combined to obtain a dual-band filter with a passband response. The filter and antenna are then integrated to provide the simultaneous filtering and radiating behavior of the subsystem, as shown in Figure 2d. As a final configuration, it can be seen in Figure 2d that the antenna and the two filters are cascaded by means of equal width (50 Ω) microstrip sections.

### 2.2. Perfect Magnetic Surface and Main Parameters of the Proposed Filtering Devices

**Figure 3:**
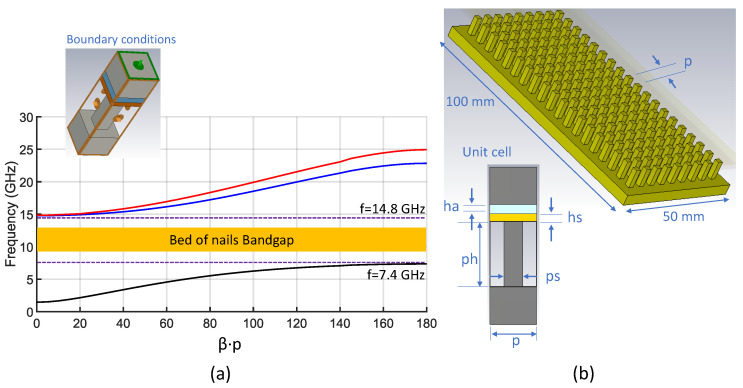
(**a**) Dispersion diagram of the bed of nails. (**b**) Finite (100 mm × 50 mm) periodic array of pins and unit cell parameters (ph = 7 mm, ps = 2 mm, p = 5 mm, ha = 1 mm, hs = 0.79 mm).

We now briefly present the artificial surface that allows the implementation of the GWG. The surface is identical to that presented in [49] and consists of a periodic array of equally spaced square metallic pins of width ps = 2 mm and height ph = 7 mm. The geometry of the array is also square and the spatial period of the pins is p = 5 mm. Figure 3b shows the unit cell used in the full-wave simulator CST Microwave Studio to calculate the dispersion diagram of the infinite periodic structure (bed of nails). In the unit cell we can also appreciate the presence of the substrate with thickness hs = 0.79 mm and the air layer with thickness ha = 1 mm. The upper part of Figure 3b shows the complete model used to simulate the finite PMC surface. The result of simulating the unit cell with the appropriate periodic boundary conditions is the GWG dispersion diagram shown in Figure 3a. The diagram shows that the propagation of electromagnetic field solutions in the bandgap is not possible. In other words, parallel plate modes cannot be excited between the start frequency $f_{star}=7.4$ GHz and the end frequency $f_{end}=14.8$ GHz of the bed of nails bandgap. The QTEM mode allows the propagation of the energy of the signal confined between the microstrip and the upper metallic plate, obtaining the self-packaging of the GWG. All this without the need for metal walls or electrical contacts, which facilitates manufacturing and assembly, reducing cost and tolerances.

**Figure 4:**
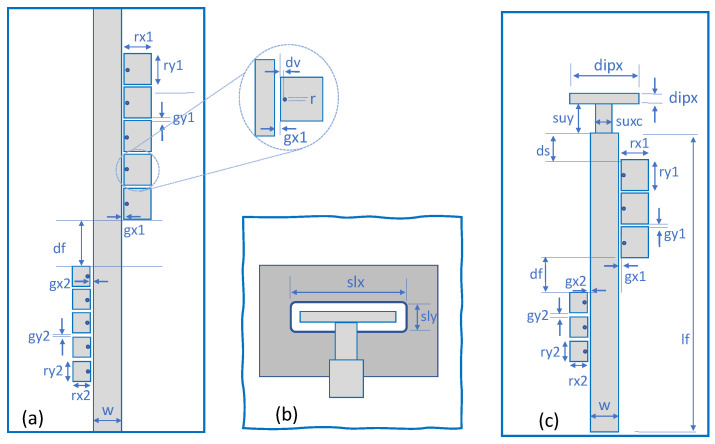
Geometry of the proposed filtering structures (microstrip printed layer) and main parameters. (**a**) Dual-band EBG filter. (**b**) Slot antenna and T-shape feeding line section. (**c**) Filtering antenna.

Once we have characterized the GWG, we consider a few basic circuits to analyze the response of the system with the variation of its elements. Figure 4 shows the main geometric parameters of the printed layers for the two different circuits (filter and antenna). Figure 4a details the variables of the dual-band EBG filter. Figure 4c describes the antenna with the filter and also allows one to identify the variables for the reference antenna. Finally, Figure 4b includes the geometric parameters of the rectangular slot antenna.

### 2.3. EBG Dual-Band Filter Analysis

**Figure 5:**
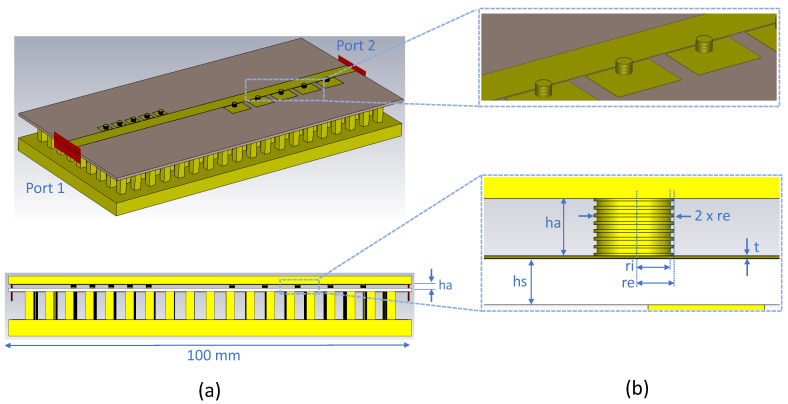
Three-dimensional geometry of EBG filter. (**a**) Perspective and side views. (**b**) Screw model zoom.

Typically, the implementation of the direct coupling method to filtering antennas requires the separate design of a PBF and a broadband antenna. In these cases, the performance of the whole system is determined to a greater extent by the filter [12]. In what follows, the method used to obtain a bandpass filter, with the appropriate individual response to be later integrated with the antenna, is described. The EBG filters that we are going to use are those proposed in [49]. They consist of a finite periodic set of mushroom-type resonators that coplanarly load the microstrip line. The resonators are formed by a rectangular patch with a small gap between the line, gx1 or gx2 in Figure 4, and connected on its side to the upper plate of the GWG by a short-circuit element. Two sets of resonators, with different patch sizes, have to be used to suppress two different frequency bands. Each of these sets corresponds to an EBG filter in Figure 1 and Figure 2 and is primarily responsible for one of the stopbands. In [49] the effect of the numerous geometric parameters that this type of filter has is studied in detail. For simplicity, except for the size of the patches, the rest of the parameters are the same for the two EBG filters. For example, the patch-line gap allows widening of the band, in this case a small gap gx1 = gx2 = 0.2 mm has been chosen to obtain a sharp selectivity. Another critical parameter in the design is the radius of the metallized via hole. In this case, it was decided to use short-circuit elements with the same radius for both filters. It would be possible to use short circuit elements of different radius, which would add one more degree of freedom to the design and would allow tuning the response. Another essential aspect is the rejection capability of the filter. As the number of resonators increases for the filter, it offers a frequency response with higher levels of rejection. To facilitate the comparison with [49], filters with five resonators were chosen, which offers a high level of rejection but a limited size. The structure chosen to analyze the combined response of the two filters is presented in Figure 5.

**Figure 6:**
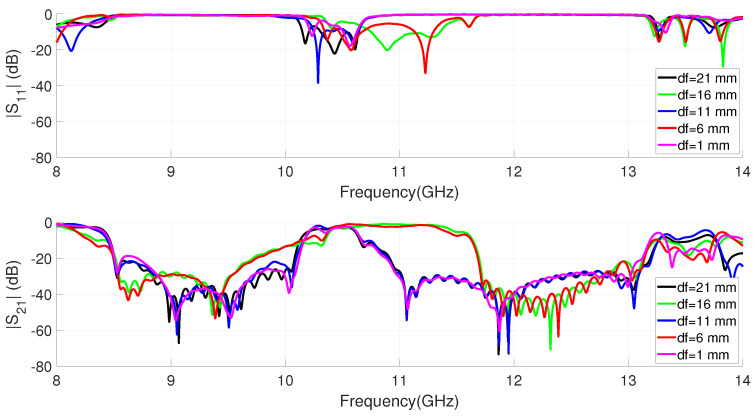
Simulated S parameters of the dual-band EBG filter for different distances (df) between filters.

A numerical analysis of the resonator sizes needed to center the rejected bands, at f = 9 GHz for filter 1 and around f = 12 GHz for filter 2, has been carried out. Although it an approximately independent response can be observed for each filter, it is evident that some coupling exists. In order to avoid the coupling, taking into account that we do not want to introduce any extra element in the circuit, it is necessary to control the combined response by means of the separation distance between filters (df in Figure 4). Figure 6 shows the S parameters simulation of the dual-band filter for different distances between the two EBG filters. It can be seen in the graph how from a certain distance the coupling level decreases, allowing a considerable passband and a flat response. Table 1 shows the resulting parameter values for the dual-band EBG filter.

**Figure 7:**
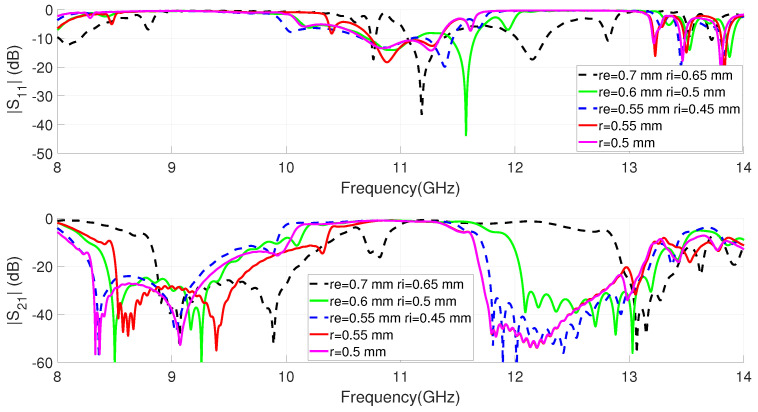
Simulated S parameters for the dual-band EBG filter for different short-circuit elements.

The response of this type of EBG filter is greatly influenced by the short circuit element. First, its position modifies the working frequency and allows compacting the resonators if it is placed on the edge of the patch. Second, the variation of the radius of the short-circuit element strongly modifies the inductance of the cell [49]. This behavior, which is clearly explained in terms of the resonator circuital model included in [21], produces significant variations in the resonance frequency and therefore in the filter band. Figure 7 shows the variation of the dual-band filter response with different shorting elements. The simulations corresponding to the metallic via modeled as a cylinder of radius r and those obtained for more complex models of screws, such as those proposed in [49], are included in the figure. Figure 5a clearly shows the position of the screws that connect the top cover of the GWG to the patch of the resonators. This configuration was already used in [49] with good experimental results. The necessary parameters for the modeling of screws of different metrics are included in Figure 5b.

Figure 7 shows how the rejected bands are located approximately at the considered frequencies to allow the propagation of the signal between 10 GHz and 11 GHz. After [49], we know that the prototypes made with screws shift their response towards lower frequencies, which implies that the effective radius of the short-circuit element is less than the nominal of the model. For this reason, to obtain a realistic model for the screw that is going to be used, the radius of the via is decreased from re = 0.7 mm and ri = 0.65 to values of re = 0.55 mm and ri = 0.45 mm. It is verified in the graph that for this size, considering the via as a cylinder of radius r = 0.5 mm, we obtain similar results. To simplify the procedure as much as possible, in what follows this value will be used in the models. In this case, it can be seen how the lower band is located according to frequencies with the measurements made in [49]. In the Figure 7, a flat passband with low insertion losses of the order of 1 dB is obtained. All materials are lossy in simulations but SMA connectors are not included. This tuned filter, with parameters given in Table 1 including the cylindrical via of radius r = 0.5 mm, is considered for integration with the antenna. Figure 7 shows two stopbands in the transmission that will be combined with the antenna, we can consider, as in [49], that a sufficient rejection level will be $|S_{12}|$ less than −20 dB. The designed filter has a lower stopband centered at f = 8.85 GHz with a fractional bandwidth of 12.4% and with a maximum rejection level of 53 dB. For the upper stopband, the center frequency is f = 12.4 GHz with 11.6% fractional bandwidth and a maximum rejection of 54 dB. Finally, from Figure 7, we can see a passband centered at f = 10.7 GHz with a 12.4% width (3 dB FBW); in this case, the minimum insertion losses are 0.98 dB at a frequency of 10.85 GHz.

### 2.4. Filtering Antenna Integrated Response

**Figure 8:**
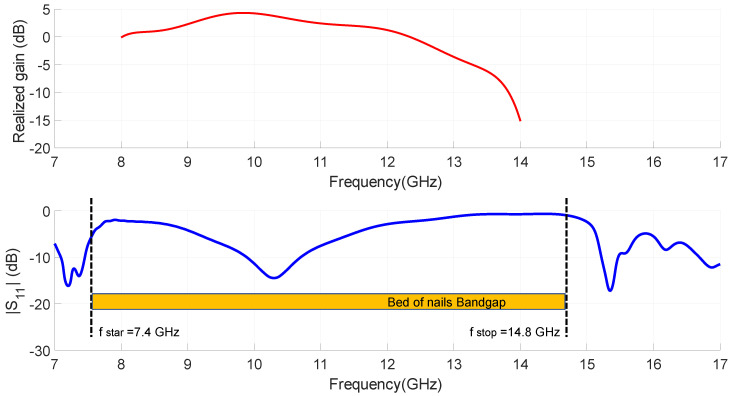
Simulated $|S_{11}|$ and realized gain (at broadside) of the reference slot antenna in IM-GWG (slx = 14.8 mm, sly = 3.25 mm, dipx = 13.8 mm and dipy = 2 mm).

According to the method used in [10], the design of a slot antenna with considerable bandwidth was employed using full wave simulations. From an impedance 50 Ω feeding microstrip line, w = 4.5 mm, a T-shape feed line section was used as shown in Figure 4b. The dipole-type end line width, dipx = 13.8 mm in Figure 4c, allows power to be coupled to the slot whose width is slx = 14.8 mm. All geometric values of the reference antenna obtained in the fitting process are included in Figure 8. The simulations of the frequency response of the slot antenna, impedance matching and broadside realized gain are included in Figure 8. We can see that $|S_{11}|<-10$ dB results in an operating band from 9.8 GHz to 10.7 GHz, corresponding to an FBW of 8.8% at a frequency of $f_{c}=10.15$ GHz. The maximum simulated realized gain for the antenna is 4.3 dBi at 9.85 GHz. We can also observe the characteristic behavior of this IM-GWG antenna. At frequencies included in the bandgap corresponding to the pins, also represented in Figure 8, the conventional response of an inverted microstrip feeding slot antenna can be seen (in this case including the self-package). Below and above this bandgap, the reflection coefficient response, in Figure 8, corresponds to the excitation of undesired modes in the parallel plate waveguide.

As discussed in the introduction, size reduction, low losses and filtering capabilities are a trend in antenna design. In our case, a trade-off between filter size, passband width and rejection level is necessary. For this reason, the filter’s antenna integration approach has two requirements. First, our objective is a very simple design that includes the possible integration of the filters without modifying the feeding lines of the antenna or the array. Second, IM-GWG antenna feeding networks are usually already complex and have space limitations, so if our goal is to integrate coplanar-EBG filters they must necessarily be as compact as possible. Two consequences follow from this argument. On the one hand, for the antenna filtering, we are going to use three-cell-based EBG-filters, as shown in Figure 1 and Figure 4c, instead of five resonators as we did when designing the filter in Section 2.3. With this, we expect a lower selectivity of the filtering, although the rejection level will be close to 20 dB [49]. On the other hand, if we do not want to introduce any extra network that matches the filter, we can basically adjust the combined filter–antenna response with the distance that separates the dual-band filter from the antenna and the separation between EBG-filters (ds and df in Figure 4c, respectively). Direct filter–antenna connection, as is our case, produces an impedance mismatch [13]. This can cause the filter’s performance to deteriorate, especially at the band edges, which in our design significantly affects the passband of the filter [25]. Usually, an extra matching network could be implemented to solve it [12], which is avoided here to make the filter integration compatible with already existing networks with strong space constraints.

**Figure 9:**
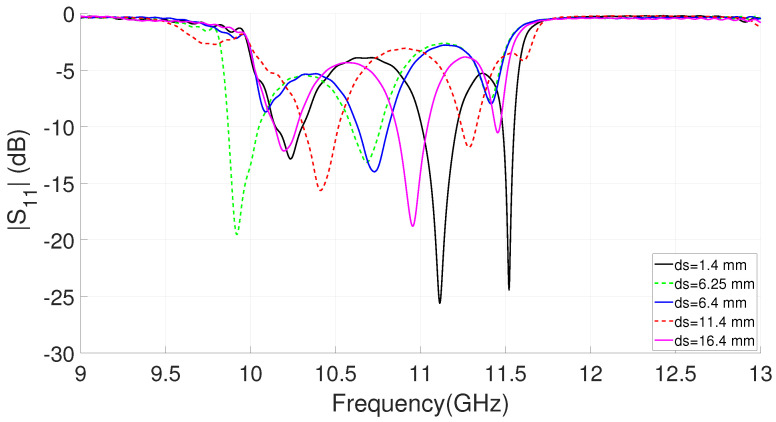
Simulated $|S_{11}|$ of analyzed filtering antenna for different lengths, (ds) in Figure 4c, between filter 1 and the radiant element (df = 7 mm).

To make the dual-band filter compact, the decrease in the distance between filter 1 and filter 2 is initially analyzed. The effect of varying the spacing between filters has already been shown in Figure 6, although in that case the filters had five resonators. After the evaluation of the coupling and even if an increase is observed, a value of df = 7 mm is taken as distance, which ensures a certain passband in the simulations. Next, a parametric study was carried out to evaluate the coupling between the antenna and the filter. The combined filter–antenna frequency response is shown in Figure 9 for different spacings, ds in Figure 4c, between the filter and the T-shape feed line of the antenna. Although a mismatch between the two devices can be seen in this case, it is partially corrected by adjusting the variable (ds) to improve the overall system. The values considered for the proposed filtering antenna are indicated in Table 2, including the adjusted distances between filters (df = 7 mm) and with the slot (ds = 6.4 mm). It should be noted that both the slot size and the rest of the parameters related to the feeding line, including the T-shape section, remain the same.

**Figure 10:**
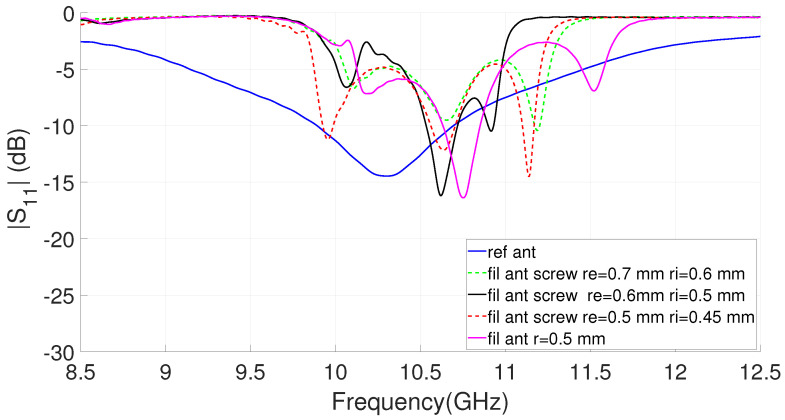
Simulated $|S_{11}|$ of analyzed filtering antenna for different short-circuit elements (see Figure 5b for the screw parameters) in comparison with the slot reference (the rest of the parameters are those shown in Table 2).

Finally, Figure 10 shows the effect on the frequency response of the antenna depending of the type of short-circuit element used. The results obtained using an M1.4 metric screw model are included together with others of slightly smaller metrics. The effectiveness of having used a simplified via model with effective radius r = 0.5 mm is verified. Figure 10 also shows the return losses for the reference antenna slot without the filter. For the case with r = 0.5 mm, the simulated impedance band for the filtering antenna ($|S_{11}|<-10$ dB) extends from 10.6 GHz to 10.85 GHz (only an FBW of 2.3%). This reveals that the integration of the EBG dual-band filter limits the bandwidth of the proposed antenna system as expected [12].

**Figure 11:**
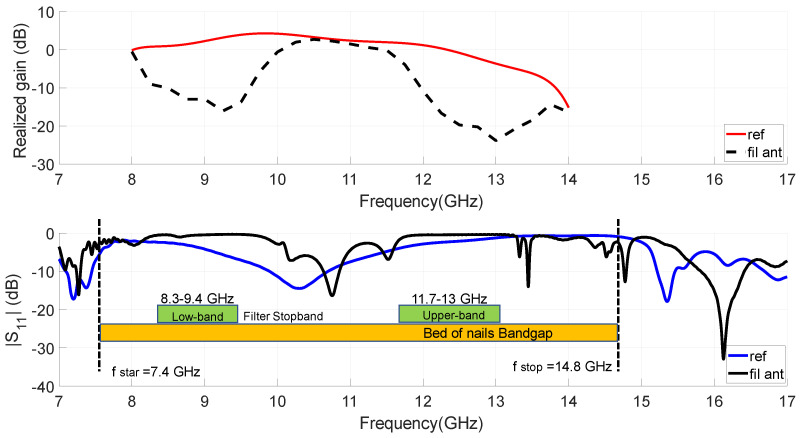
Simulated $|S_{11}|$ and broadside realized gain (at broadside) of the proposed IM-GWG filtering antenna in comparison with the slot reference.

The performance comparison between the filtering antenna and the reference, both in impedance and radiation, is depicted in Figure 11. From the impedance response, the greater frequency selectivity that the filtering antenna has with respect to the reference case is verified. The realized gain for the proposed antenna reaches a maximum value of 2.8 dBi at a frequency of 10.5 GHz. If we relax the matching level condition, $|S_{11}|<-6$ dB, the working band of the proposed antenna would be an FBW of 7.6% with central frequency at $f_{c}=10.5$ GHz. It could be seen in Figure 11 that the gain values obtained in this passband by the filtering antenna are similar to those of the reference one, except in the lower part where they are slightly lower. From Figure 11, we can draw conclusions concerning the design filtering operation. Compared to the slot reference, the proposed antenna effectively suppresses unwanted signals out of the band. The rejection level between both radiators reaches more than 10 dB between 8.75 and 9.75 GHz for the lower band and between 12.1 and 13.6 GHz for the upper band case. These rejected bands reasonably coincide with the stopbands for the designed filter in Section 2.3, also included in the graph, as shown in Figure 11. This is consistent with the direct integration method used, which initially considers that the responses of the filter and of the antenna, are independent.

**Figure 12:**
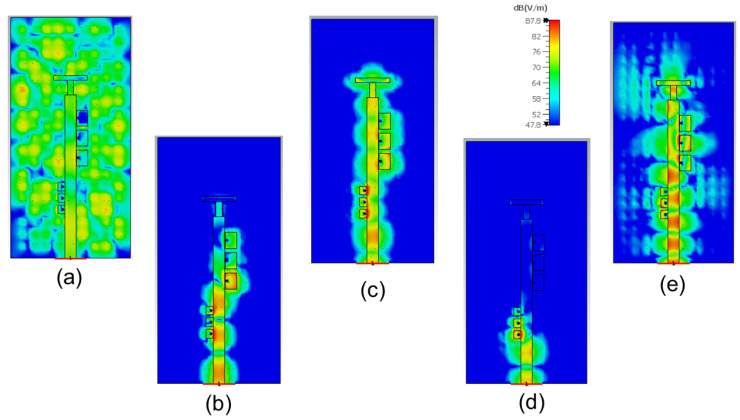
Electric field distribution $|E_{z}|$ for the filtering antenna at frequencies: (**a**) f = 7 GHz (**b**) f = 9 GHz (**c**) f = 10.5 GHz (**d**) f = 12.5 GHz and (**e**) f = 15 GHz.

In order to clarify the operational mechanisms associated with the filtering slot antenna over a wide frequency range, the calculated electric field distributions for different frequencies are illustrated in Figure 12. As predicted by the fundamentals of the GWG, the parallel plate waveguide excites modes that can propagate through the structure up to the frequency at which the artificial surface begins to behave like a PMC. As Figure 3 shows, this frequency is in our case f = 7.8 GHz. This is clear also from the return loss results obtained for the filter and the filtering antenna, as shown in Figure 11. The field distribution shown in Figure 12a at 7 GHz represents the propagation of these unwanted modes in the parallel plate waveguide. Something similar is also observed for the field distribution at 15 GHz included in the Figure 12e, since this frequency is located above the bandgap of the GWG structure. In the other three field distributions included in Figure 12, the GWG is working on the bed of nails bandgap. At the working frequency (f = 10.5 GHz in Figure 12c), the filtering antenna is excited in a similar way to the reference antenna since it corresponds to the passband of the integrated filter. While for the frequencies of 9 GHz and 12.5 GHz, included in Figure 12b,d, the band notches produced by filter 1 and filter 2, respectively, reject the energy propagated by the IM-GWG line.

**Figure 13:**
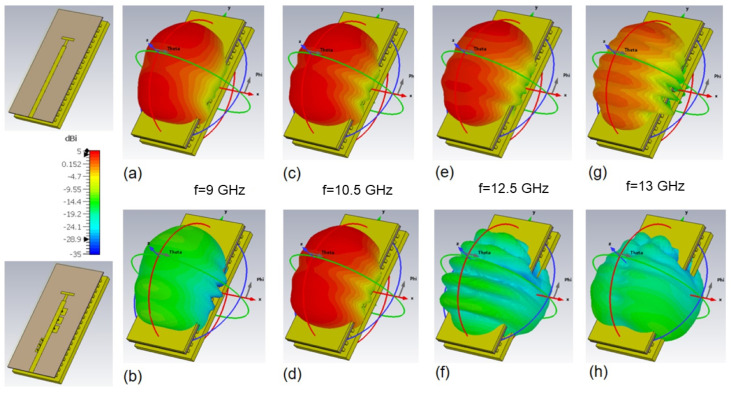
Simulated 3D radiation patterns for the reference and filtering antenna at different frequencies: (**a**) reference at f = 9 GHz (**b**) filtering at f = 9 GHz (**c**) reference at f = 10.5 GHz (**d**) filtering at f = 10.5 GHz (**e**) reference at f = 12.5 GHz (**f**) filtering at f = 12.5 GHz (**g**) reference at f = 13 GHz and (**h**) filtering at f = 13 GHz.

The simulated 3D radiation patterns for the reference slot and the filtering antenna are compared in Figure 13 for 9, 10.5, 12.5 and 13 GHz frequencies, all included in the bandgap of the GWG parallel plate. It can be concluded from Figure 13 that the radiation patterns at f = 10.5 GHz are similar, both in shape and gain levels, for both antennas. That is, the integrated antenna preserves the power transmission, as observed in Figure 12c. In the bands rejected by the dual-band filter, at 9 GHz for filter 1 and at 12.5 and 13 GHz for filter 2, the filtering antenna’s radiation patterns maintain symmetry but theirs power are significantly reduced by approximately 12 dB and 20 dB, respectively.

**Figure 14:**
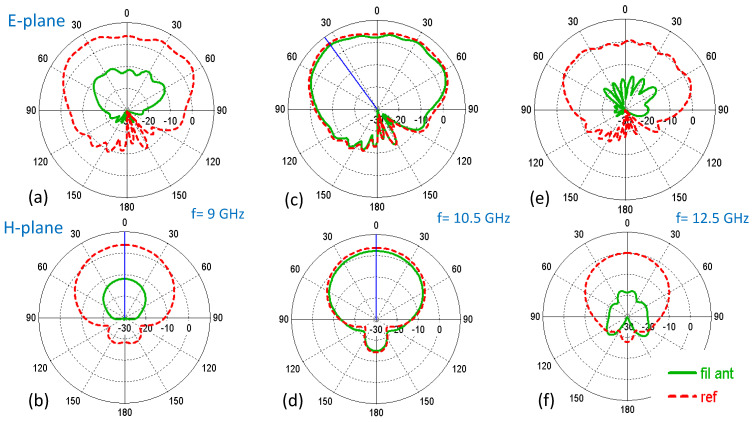
Simulated radiation patterns (realized gain) of the filtering antenna and the reference slot at different frequencies: (**a**) E-plane at f = 9 GHz (**b**) H-plane at f = 9 GHz (**c**) E-plane at f = 10.5 GHz (**d**) H-plane at f = 10.5 GHz (**e**) E-plane at f = 12.5 GHz and (**f**) H-plane at f = 12.5 GHz.

To facilitate the comparison between the proposed antenna and the slot antenna without the filter, the polar radiation patterns for the E and H planes of the antenna are included in Figure 14. As expected, the patterns at the operating frequency for both planes are very similar, as shown in Figure 14c,d, while at the rejection frequencies produced by the EBG-filters there is a very significant decrease in the radiated power as observed in Figure 14a,b and Figure 14e,f for the lower f = 9 GHz and higher f = 12.5 GHz filter frequencies, respectively.

## 3. Experimental Results and Discussion

The manufactured prototypes and the main experimental results achieved are described in this section. There are three devices that have been manufactured and that use the same bed of nails: the dual-band filter that integrates the two EBG stopband filters, the slot antenna that will be used as a reference and the integrated filtering antenna. At the end of the section, experimental results and important aspects in the design and operation of the system will be discussed.

### 3.1. Filter Measurements

**Figure 15:**
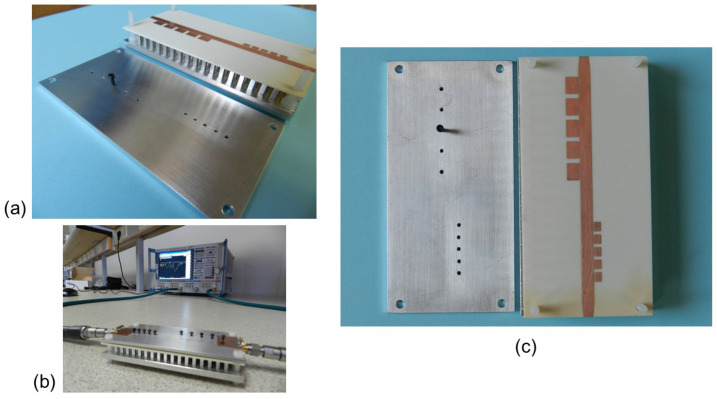
Photograph of the fabricated dual-band filter: (**a**) Perspective view (unstacked layers). (**b**) Filter test setup. (**c**) Front view with the detail of the printed layer with the resonators and the threads for the screws on the top plate.

**Figure 16:**
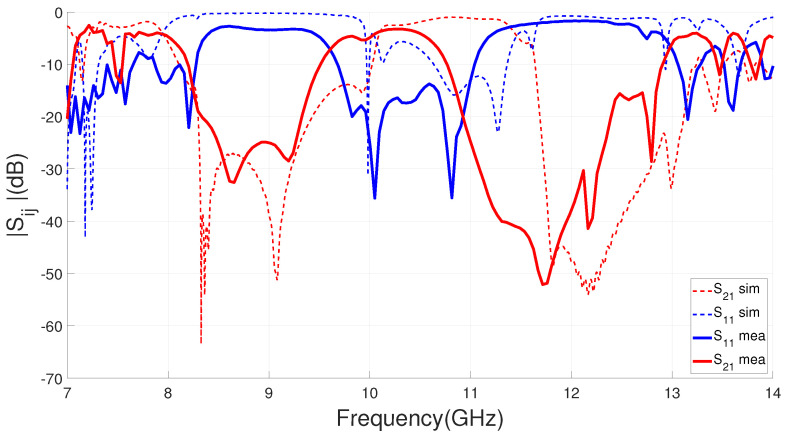
Measured versus simulated S parameters for the designed IM-GWG dual-band filter with geometrical parameters included in Table 1.

The designed dual-band filter with parameters included in Table 1 has been manufactured for characterizing its scattering parameters. The filter prototype and experimental setup are included in Figure 15. Identical screws to those used in [49] of metric M1.4 have been used as short-circuit elements in both filters, as shown in Figure 15. As explained in [49], since the filter is designed to be integrated into the antenna, it is not relevant to know the exact level of losses that could be achieved by the device. Therefore, the measurements are those directly obtained from the vector network analyzer, without using any TRL calibration kit and including the effect of the connectors. The solderings can be seen in the photograph included in Figure 15b and are those made in the laboratory as a proof of concept. To allow for soldering, a small taper on the line is observed in Figure 15c, right at the junctions with SMA connectors that have no effect on the filter’s S-parameters. Figure 16 shows the simulated and measured filter response from 7 to 14 GHz. In terms of selectivity and rejection levels, the overall measured result agrees well with the simulations, showing two stopbands centered at 8.8 GHz and 11.7 GHz. Out of band frequencies of 8.3–9.4 GHz and 10.9–12.4 GHz are significantly suppressed by more than 20 dB, as Figure 16 shows. As we will see later, we want to avoid unwanted radiation in these bands. Table 3 includes a summary of the comparison between simulations and measurements for the filter.

Compared to the simulations, the upper stopband of the filter is shifted to lower frequencies, as shown in Table 3. Figure 16 also shows a shift in the passband towards lower frequencies for the filter measurements. These discrepancies could have some physical reasons, including manufacturing tolerances, misalignments or mounting issues (including the soldering of the SMA connectors to the GWG parallel plate). However, these discrepancies may be due to modeling errors. For example, they could be due to small errors in the simulated values of the permittivity and the level of losses. Finally, they may be due to the difficulty of modeling the short-circuit elements in a wide frequency range, which implies an inaccuracy in the via modeling for higher frequencies.

### 3.2. Filtering Antenna Measurements

To confirm the design procedure, the proposed IM-GWG filtering antenna is fabricated for experimental validation. In order to quantify the improvement produce by EBG filtering structure integration, the slot antenna was also manufactured as a reference.

**Figure 17:**
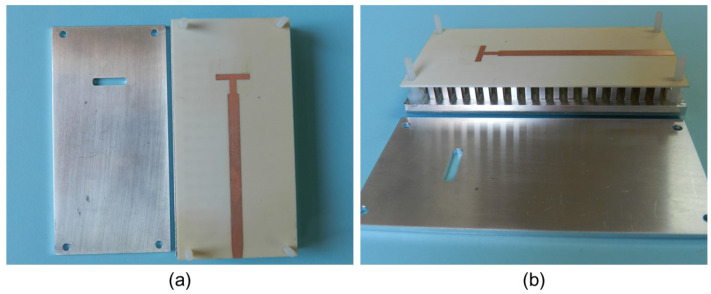
Photograph of the fabricated slot reference antenna: (**a**) Front view of the slot layer and the microstrip feeding line with the T-shape feeding line section. (**b**) Side view of the GWG antenna with the top cover plate removed.

**Figure 18:**
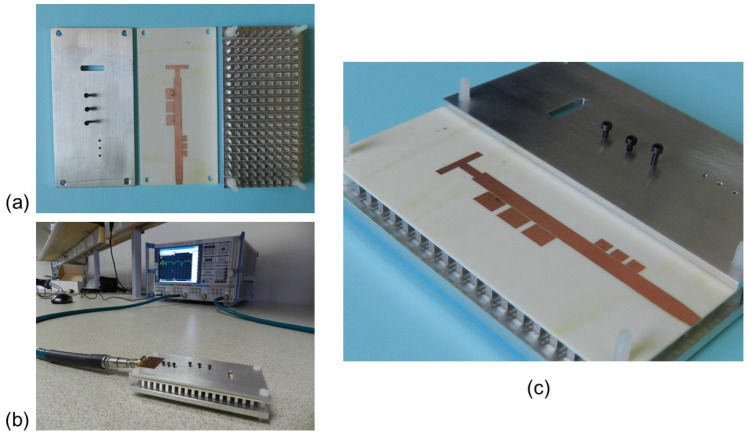
Photograph of the fabricated filtering antenna: (**a**) Front view of the layers (unstacked). (**b**) $|S_{11}|$ antenna test setup. (**c**) Perspective view with the detail of the printed layer with the resonators and the slot feeder and the threads for the screws on the top plate.

Figure 17 includes two photographs of the manufactured slot reference antenna prototype. Figure 17a shows the 2 mm thick aluminum metal cover manufactured according to the model included in Figure 1. The cover includes the slot, which for practical reasons had to be milled with a rectangular geometry but slightly rounded at the corners as seen in the image. You can also see the substrate with the printed feeding line that incorporates the T-shape section and the taper in the line to solder the SMA connector. Finally, the multilayer configuration is shown in Figure 17b, where you can see the bed of nails, the position of the substrate supported by the pins and the thickness of the slot layer. The fabricated prototype of the proposed filtering antenna is shown in Figure 18. All the values of its geometric parameters are included in Table 2. In the first image, you can see the three unstacked layers needed for the IM-GWG device (Figure 18a). The experimental setup for the impedance measurement is shown in Figure 18b, in which the soldered SMA connector can be seen. Finally, the detail of the printed patches of the resonators and the M1.4 metric screws equal to those used for the dual-band filter in Section 3.1 are shown in Figure 18c.

**Figure 19:**
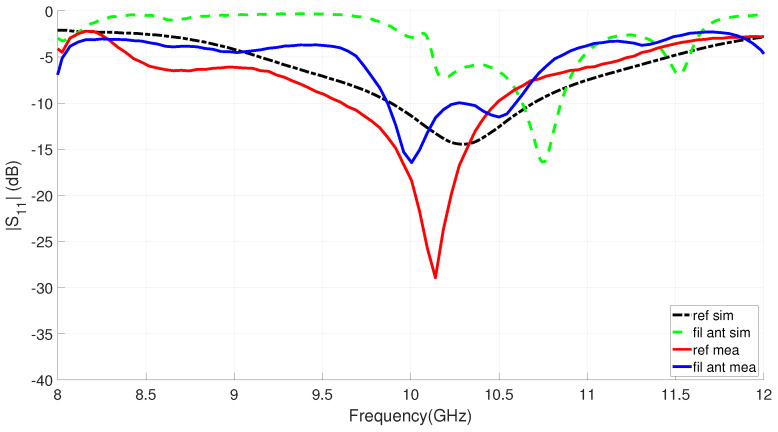
Measured and simulated frequency response $|S_{11}|$ of the proposed IM-GWG filtering antenna compared to the reference slot antenna.

The simulated and measured reflection coefficients for the filtering and reference antennas are shown in Figure 19. A reasonable agreement is observed between the measurements and the simulations that validate the analysis performed. For the proposed filtering antenna, we can observe a $|S_{11}|$< −10 dB operating band from 9.85 to 10.6 GHz, which means a 7.3% FBW at a 10.23 GHz central frequency. In the case of the slot reference antenna, the impedance matching band is centered at 10.05 GHz and reaches an FBW of 9%. As expected, on the one hand, the integration of the filter produces an increase in the selectivity of its frequency response. While on the other hand, the impedance matching of the proposed antenna is mainly affected by the PBF reducing its bandwidth, as shown in Figure 19. Finally, the discrepancy observed in Figure 19, between the simulation and the measurement for the reference antenna suggests some possible small errors due to manufacturing tolerances and in the modeling of the dielectric constants of the substrate. It is reasonable to suspect that these small errors also exist for the measurement–simulation comparison of the filtering antenna.

**Figure 20:**
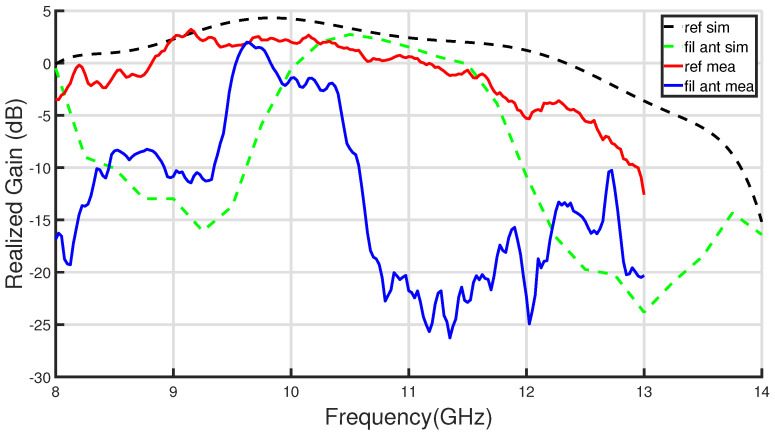
Comparison of measured and simulated broadside gain frequency response between the proposed IM-GWG filtering antenna and the reference slot antenna.

To verify the radiant and filtering functions of the proposed design, the filtering antenna has been measured together with the reference one in the anechoic chamber of our laboratory. Figure 20 depicts the comparison of broadside realized gain response versus frequency between the proposed antenna and the reference antenna.The in-band measured peaks of the realized gain are 2.7 dBi at 10.15 GHz and 2.1 dBi at 9.62 GHz for the reference and filtering antenna, respectively. These values have been obtained by comparing with a standard horn antenna. In relation to the filtering antenna, passband performance is clearly observed between 9.48 GHz and 10.4 GHz, while in the lower and higher bands there is a high rejection level in the radiated power. The radiation results obtained for the antenna in comparison with the simulations are summarized in Table 4. The antenna measurements present an overall good agreement with simulations included in Figure 20, demonstrating the proposed concept of filtering antenna and can be verified in Table 4. The gain levels are similar and the filtering behavior of the simulated antenna is clearly confirmed by the experiment. Regarding the bands filtered by the antenna, on one hand, the lower band reaches rejection levels between 10 and 14 dB, which are lower than simulated. For the upper band, both the rejection levels and the filtering bandwidth (see Rj. 10 dB BW in Table 4) are very similar. In the measurement of the filtering antenna, a shift in its frequency response is observed, which is greater for the passband and for the upper filtered band. This upper band frequency shifting was already reported in the dual-band EBG response shown in Figure 16. So the discrepancy is not due to the integration of the filter with the antenna. In part, the difference is due to the difficulty of modeling correctly and simultaneously the effect of the short-circuit elements in the two stopbands of the filters. One possibility that has not been addressed is to consider, in each band, a different adjustment of the effective radius of the screws and evaluate it experimentally before design.

### 3.3. Discussion

The measurements show that in the proposed filter–antenna subsystem, it is possible to obtain at the same time an efficient transmission in the passband and improved out-of-band with high levels of rejection.

Based on the direct coupling method, a design guide is described as follows.

1. The dispersion diagram for the bed of nails is simulated, obtaining the periodicity and height and width of the metallic pins to achieve a bandgap that includes the necessary operating band of the GWG. The pins must have a height close to λ/4 to achieve the behavior as PMC. The air gap is selected according to the bandwidth and transmission power needed in the system, always maintaining a distance significantly smaller than λ/4 between the substrate and the top cover of the GWG parallel plate.
2. The slot antenna is designed by adjusting the dimensions of the slot and the printed excitation dipole. Finally, the other parameters of the T-shape feed line section are optimized to maximize the impedance bandwidth.
3. The filter is designed using two sets of EBG-resonators, adjusting the sizes of the patches and selecting the radius of the short circuit elements. Previously, the coplanar gap between the line and the resonators was selected based on the required filtering bandwidth. The two sets of resonators are coplanarly placed on each side of the feeding line a certain distance apart. This distance is adjusted by controlling the coupling between the filters. This determines the passband of the filter, affecting its bandwidth, its flatness and the losses of the integrated system.
4. The dual-band filter is integrated into the antenna’s feeding line, tuning the distance between the filter and the T-shape line section to control the coupling between the antenna and the filter. The impedance response of the integrated antenna is optimized and the necessary radiation performance for the transmitted band and the rejected bands is verified. Finally, the stability of the in-band radiation pattern for the filtering antenna is checked.

To the best of our knowledge, this filtering antenna is the first to be experimentally demonstrated in IM-GWG technology with good radiation performance and simultaneous filtering functionalities. The EBG filters used based on [49] are easy to design and adjust and have many parameters that make their tuning flexible in terms of defining their frequency selectivity. For this reason, the proposed configuration opens up the possibility of integrating GWG antennas and arrays with a similar filtering topology. This would have the advantage of adding new functions to the antennas such as reducing unwanted radiation or eliminating interfering power, obtaining high isolation between ports in multiband systems or improving the selectivity of the system at the edge bands.

**Figure 21:**
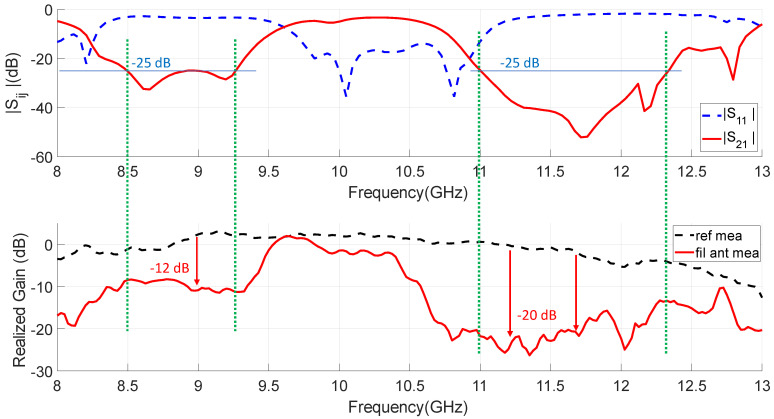
Comparison of the measurements obtained for the stand alone EBG filter frequency response (based on five-cell EBG) and the broadside realized gain of the proposed integrated IM-GWG filtering antenna (based on three-cell EBG).

As can be seen in Figure 21, the measured transmission response of the dual-band filter associated with the measured realized gain at broadside for the proposed filtering antenna are both plotted for comparison. It is found that in the bands with a high level of filter rejection it is possible to reduce the power radiated by the antenna in a very effective way (more than 12 dB in the lower band and more than 20 dB in the upper one). The selectivity in the antenna response is lower than in the filter, which is consistent with the simple direct integration method applied in the design and with the fact that the EBG filters of the antenna are based on three resonators. As discussed in [49], these filters are very compact compared to other similar state-of-the-art filters. It is demonstrated that this type of EBG filter can be integrated, with minimal complexity in the feeding network, to add filtering functions in GWG antennas.

**Figure 22:**
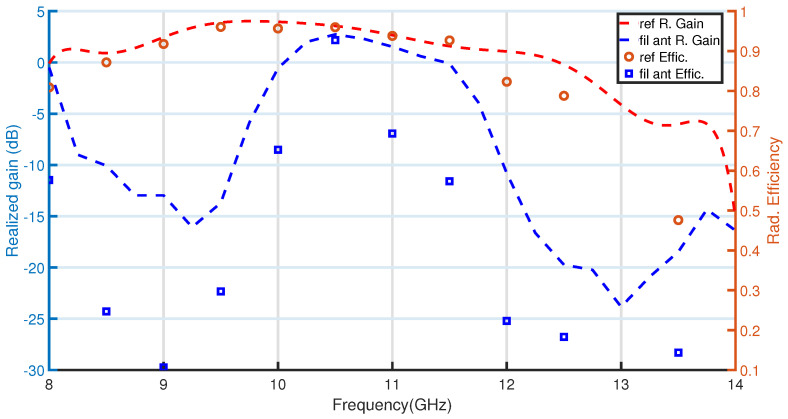
Simulated radiation efficiency and antenna realized gain of the filtering antenna in comparison with the reference slot.

In addition, the simulated radiation efficiency for the filtering antenna and the reference antenna is also given in Figure 22. It is observed that the efficiencies at the lower and upper stopbands are lower than 12% and 19%, respectively. This proves the effective rejection produced by the EBG-filters compared to the efficiencies of the slot antenna at these frequencies, which are 92% at f = 9 GHz and 79% at f = 12.5 GHz. It also reveals that radiation is suppressed in all directions of space not only at boresight. In addition, within the working band, the filtering antenna efficiency is 92% at a frequency of 10.5 GHz, which shows good in-band radiation. Figure 22 shows that the efficiency for the proposed antenna, with the resonators, is slightly lower than the reference antenna which is 96% at the frequency of 10.5 GHz.

In order to evaluate the characteristics obtained by the proposed IM-GWG antenna, Table 5 shows a comparison with other published filtering antenna designs. The list includes different technologies and briefly describes the method used to filter. It also includes the type of radiating element used as antenna, slots or patches and its basic antenna parameters. Table 5 also gives the rejection level achieved in antennas with dual band filtering; both data are included and sometimes the isolation level obtained in diplexed systems is provided. Finally, the level of complexity of the filtering antenna is illustrated. The degree of complexity increases as the antenna uses only one cascading filter, an extra matching circuit, different filtering elements, several layers, etc. From Table 5, we can conclude that on the one hand the results obtained in our design are competitive in terms of bandwidth, gain and rejection level; and on the other hand, this work implements a simpler design method that allows integrating filtering functions into IM-GWG antennas, including all the advantages of GWG technology. The subsystem described here can be useful in the first place to mitigate interference in the X-band, which, in addition to communications applications, is widely used for radar and satellite systems. Furthermore, if the design is scaled in frequency, it can be used to reduce interference or to isolate frequencies in RF front-ends of mmWaves in which losses and integration are critical aspects.

## 4. Conclusions

A filtering antenna in inverted microstrip Gap Waveguide Technology has been proposed, fabricated and tested. The design is simple; it consists of the direct integration of two compact coplanar EBG-filters that do not require any modification in the feeding line or any extra circuitry. The filtering antenna concept in GWG has been experimentally verified with simultaneous radiation and filtering functions. The measured results have shown good performance in terms of realized gain (2.1 dBi) and impedance matching (7.3%). The impedance matching of the proposed antenna is mainly affected by the filtering structures. The integrated antenna effectively suppresses unwanted signals out of the band, reaching rejection levels of 14.7 dB and 25.7 dB in the lower and upper band, respectively. The GWG filtering antenna is capable of effectively reducing RF front-end losses, of including self-package and of reducing its overall size, eliminating the filter.

## Figures and Tables

**Table 1:** Parameters of the dual-band EBG IM-GWG filter.

**Parameter**	rx1	ry1	gy1	rx2	ry2	gy2
**value (mm)**	4.8	6.5	1.75	2.8	3.5	1.25
**Parameter**	gx1	gx2	dv	df	r	ha
**value (mm)**	0.2	0.2	0.35	16	0.5	1

**Table 2:** Parameters of the IM-GWG filtering antenna.

**Parameter**	slx	sly	dipx	dipy	suy	sux	ds
**value (mm)**	14.8	3.25	13.8	2	6.1	2.5	6.4
**Parameter**	rx1	ry1	gy1	rx2	ry2	gy2	lf
**value (mm)**	4.8	6.5	1.75	2.8	3.5	1.25	67.9
**Parameter**	gx1	gx2	dv	df	r	ha	hs
**value (mm)**	0.2	0.2	0.35	7	0.5	1	0.79

**Table 3:** Simulated and measured IM-GWG EBG dual-band filter comparison performance.

Dual-Band Filter Operation									
**frequency band**		unwanted. filter 1			operation			unwanted. filter 2	
**function**		Stopband			Passband			Stopband	
**response**	f_c (GHz)	−20 dB FBW (%)	Max. Rj Lev (dB)	f_c (GHz)	−3 dB FBW (%)	Min. IL (dB)	f_c (GHz)	−20 dB FBW (%)	Max. Rj Lev (dB)
**simulated**	8.85	12.4	53	10.7	12.4	0.98	12.4	11.6	54
**measured**	8.8	11.7	32.6	10.2	9.7	3.1	11.7	12.7	52.1

**Table 4:** Simulated and measured IM-GWG Filtering antenna comparison performance.

Filtering Antenna Operation											
**frequency band**		operation				filter 1			filter 2		
**function**		radiation				rejection			rejection		
	f_c	RL. 10 dB BW	f_c	RL. 6 dB BW	Peak R.Gain	f_s	Rj. 10 dB BW	Max. Rj	f_s	Rj. 10 dB BW	Max. Rj
	(GHz)	(%)	(GHz)	(%)	(dBi)	(GHz)	(%)	(dB)	(GHz)	(%)	(dB)
**sim**	10.7	2.3	10.5	7.6	2.8	9.2	11.4	19.3	12.8	11.7	20.2
**mea**	10.23	7.3	10.3	10.3	2.1	9.1	5.5	14.7	11.4	15.8	25.7

**Table 5:** Performance comparison with other reported filtering antennas.

Ref.	Technology	Radiator	Filtering Method	Freq. (GHz)	Bandwidth (%)	Gain (dBi)	Rejection Level (dB)	Complexity
[17]	Microstrip	Patch	EBG filter	2.1/2.45	3.8/3.8	7.3/8.2	26/13	Low
[31]	SIW	Slot	slot-loaded SIW cavity	4.3/5.02	2.4/6	5.94/6.45	25 (isolation)	High
[24]	SIW	Patch	SIW cavity	4.25/5.11	2.8/4.7	7.93/7.44	25 (isolation)	High
[29]	Microstrip	Patch (array)	Coupled resonator	2.38	5.6	9.7	22	High
[39]	Microstrip	Patch	Slot + strip + pins	3.4	23.5	6.8	17	High
[21]	Waveguide	Slot (array)	EBG surface	12.5	4	13.6	30	Medium
[43]	GWG (Groove)	Slot (array)	Diplexer	28.21/29.21	2.3/2.2	31/31.5	45 (isolation)	High
[42]	GWG (Groove)	Slot (array)	Diplexer	73.5/83.5	6.8/6	24/21	50 (isolation)	High
This work	GWG (IM)	Slot	EBG filter	10.23	7.3	2.1	14.7/25.7	Low

## Data Availability

Not applicable.

## Abbreviations

The following abbreviations are used in this manuscript:

BSF	Band stop filter
BPF	Band pass filter
EBG	Electromagnetic bandgap structures
FBW	Fractional bandwidth
GWG	Gap Waveguide Technology
IM-GWG	Inverted microstrip Gap Waveguide Technology
PCB	Printed circuit board
PEC	Perfect electric conductor
PMC	Perfect magnetic conductor
QTEM	Quasi transverse electromagnetic
RF	Radio frequency
SIW	Substrate integrated waveguide
